# Characterization of a New HIV-1 Second-Generation Circulating Recombinant Form CRF173_63A6 in the Jewish Autonomous Region of Russia

**DOI:** 10.3390/pathogens14090836

**Published:** 2025-08-22

**Authors:** Vasiliy E. Ekushov, Maksim R. Halikov, Alexei V. Totmenin, Mariya E. Antonets, Tatyana V. Tregubchak, Andrey I. Murzin, Marina N. Pavlova, Anastasia M. Troianova, Tatyana P. Adusheva, Svetlana N. Beniova, Alexandra S. Ermolitskaya, Irina S. Gorelova, Alexander P. Agafonov, Natalya M. Gashnikova

**Affiliations:** 1 State Research Center of Virology and Biotechnology VECTOR, 630559 Koltsovo, Russia; halikov_mr@vector.nsc.ru (M.R.H.); starchevskayamaria@mail.ru (M.E.A.); tregubchak_tv@vector.nsc.ru (T.V.T.); ngash@vector.nsc.ru (N.M.G.); 2 Regional State Budgetary Healthcare Organization “Center for the Prevention and Control of AIDS” of the Jewish Autonomous Region, 679000 Birobidzhan, Russia; m.podruga@yandex.ru (M.N.P.); sa-3107@yandex.ru (A.M.T.); tatyana.aduscheva@yandex.ru (T.P.A.); 3 Regional Clinical Hospital No 2, 690011 Vladivostok, Russia; snbeniova@mail.ru (S.N.B.); erasvet27@yandex.ru (A.S.E.); gorelova_ira@mail.ru (I.S.G.)

**Keywords:** HIV-1, molecular epidemiology, recombination, genetic variation

## Abstract

Studies of HIV-1 molecular epidemiology describe significant differences in HIV infection spread across geographical areas. We examined 80 HIV-1 samples from the Jewish Autonomous Region of Russia in 2024. HIV-1 genome sequences for 12 samples revealed a novel HIV-1 called CRF173_63A6. HIV-1 CRF173_63A6 was found to have arisen through recombination between a specific Russian A6 subtype and the recombinant virus CRF63_02A6, which is responsible for the PWID-associated HIV outbreak in the Siberian region of Russia. Phylogenetic analysis of pol sequences previously deposited in Genbank showed that the CRF173_63A6 samples we described are grouped into a common phylogenetic cluster that includes 54 HIV-1 samples isolated in the JAR and other areas of the Russian Far East, indicating a wide distribution of this virus genovariant. This study once again proves the significant contribution of the key PWID group not only to the development of local Russian HIV epidemics, but also to the change in the characteristics of the circulating virus population.

## 1. Introduction

Studies of HIV molecular epidemiology in Russia describe significant differences in HIV infection spread across geographical areas: while the specific Russian subtype A6 HIV-1 continues to dominate in the Central and Western parts of the country [1,2,3], the CRF63_02A6 virus predominates in the regions of Siberia, where the HIV infection rate exceeds the national average [4,5,6]. Research into the genetic diversity of HIV in the Russian Far East indicates the development of separate internal epidemics, which is associated with the remoteness and geographic isolation of a number of territories [7,8,9]. The Jewish Autonomous Region (JAR), part of the Far Eastern Federal District of Russia, is one of the most sparsely populated areas of the country, with 146,000 residents. According to the Regional AIDS Center, a total of 487 cases of HIV infection are registered in the JAR. In recent years, various forms of recombinant HIV have contributed more to the development of the local epidemic in the Russian Far East [10,11]. Based on the analysis of complete viral genomes, our study identifies and describes a new second-generation circulating recombinant form of HIV-1 that has spread in the JAR.

## 2. Materials and Methods

### 2.1. Sample Source

Plasma samples were collected from patients at the AIDS Prevention and Control Center, Birobidzhan, Jewish Autonomous Region and Regional clinical hospital No 2, AIDS Prevention and Control Center, Vladivostok, Primorsky Krai, in 2023–2024.

### 2.2. Ethics

The study was conducted in accordance with the Helsinki Declaration, and the protocol was approved by the Ethics Committee of the “JAR AIDS Prevention and Control Center” (Protocol No. 2 of 27 January 2021). All participants provided informed consent for the collection and subsequent analysis of their samples.

### 2.3. Sequence Analysis

The GenBank accession numbers for the NFLG sequences of the studied isolates are PQ523366-PQ523377 and PQ585408-PQ585414. The HIV-1 sequences we obtained were compared with reference sequences of different subtypes and recombinant forms from the international database GenBank in the MEGA11 and AliView programs [12,13]. Multiple alignment was performed using the MAFFT version 7.526 (RIMD) program with standard settings [14].

The maximum likelihood phylogenetic tree was created using the online resource IQ-TREE v1.6.12 with a bootstrap of 1000 repeats based on the GTR + I + G substitution model, and bootstrap analysis was used to evaluate the topology [15]. The phylogenetic tree was visualized using the iTOL toolkit [16].

RIP, jpHMM, and Bootscan assays were used to determine the recombination structures of viruses [17,18,19]. The following settings were used to perform the Bootscan analysis: window size 400 bp and step size 50 bp. The following reference subtypes of HIV-1 were used as comparison sequences: A6, CRF63_02A6, and a representative sequence of subtype B. Recombinant mosaic map of CRF173_63A6 was generated using the Recombinant HIV-1 Drawing Tool (https://www.hiv.lanl.gov/content/sequence/DRAW_CRF/recom_mapper.html, accessed on 26 September 2024).

The tMRCA was evaluated using the Nextstrain software package [20].

## 3. Results

Analysis of 80 HIV-1 samples, which we isolated from patients at the JAR AIDS Centre in 2024, revealed the following distribution of HIV genovariants: 51.3% belonged to subtype A6; 10.0% to subtype B; 5.0% to CRF02_AG; 6.3% to CRF63_02A6; and 1.3% each to A1, G, and URF_A6C. A group of 19 samples (23.8%) formed a separate HIV-1 phylogenetic cluster.

Near-full-length HIV-1 genome (NFLG) sequences for 12 samples from the established phylogenetic group of viruses were successfully obtained (the details of the patients’ demographics and clinical characteristics can be found in Table S1). Additionally, the sequences of HIV isolated from residents of the Primorsky Territory and the JAR, related to the genetic variants A6 (14 samples) and CRF63_02A6 (5 samples), were used to analyze the genome structure of the studied viruses.

**Figure 1:**
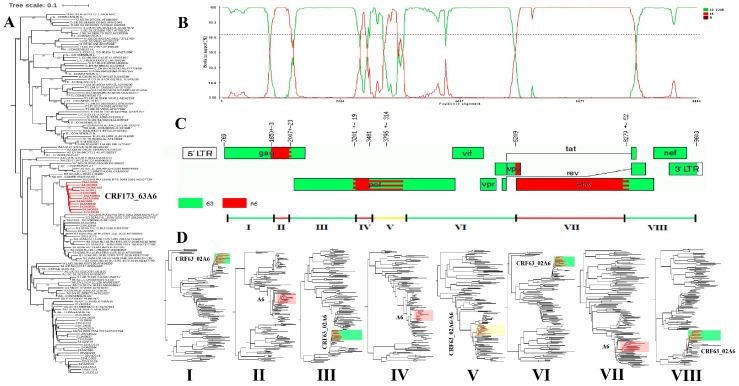
Maximum likelihood phylogenetic tree of NGFL sequences from 12 new CRF173_63A6 recombinants found in the regions of the JAR and Primorsky Krai (**A**). The color codes for the different subtypes are as follows: A6 is red and CRF63_02A6 is green (**B**–**D**). Recombinant mosaic map of CRF173_63A6 (**C**). Phylogenetic trees of the CRF173_63A6 subregion (**D**).

Phylogenetic analysis showed that the 12 studied HIV-1 samples, which were subsequently assigned the name CRF173_63A6, grouped outside of any known HIV-1 or CRF subtype and formed a separate monophyletic branch with a bootstrap value of 100% (Figure 1), indicating their origin from a single ancestor.

The 12 CRF173_63A6 genomes that we described had identical recombination profiles. Seven recombination breakpoints were identified in the genome; these were located in the gag (two breakpoints), pol (three breakpoints), vpu (one breakpoint), and env (one breakpoint) genes. All strains had these recombination breakpoints in common. Subregional phylogenetic analysis of eight genomic segments was conducted to study their probable parental lines. Phylogenetic analysis of subregions I, III, VI, and VIII confirmed their relationship with CRF63_02A6 viruses. Segments II, IV, and VII of the studied CRF173_63A6 were related to the phylogenetic group of the HIV-1 subtype A6 circulating in Russia. Fragment V does not have precise coordinates of the recombination break and is located between subtypes A6 and CRF63_02A6 on the phylogenetic tree.

Effectively, HIV-1 CRF173_63A6 resulted from recombination between CRF63_02A6 and A6 with seven breakpoints delimiting four CRF63_02A6 fragments, three fragments of different lengths of the A6 subtype, and one fragment with no clear boundaries between the subtypes. Figure 1 provides the genomic subregion and genome coordinates according to HXB2.

To understand the origin of the CRF173_63A6 variant, an additional tMRCA assessment was performed. As shown in Figure S1, CRF63_02A6 and CRF173_63A6 HIV-1 originated from the same ancestor, and the CRF173_63A6 variant we describe began to spread in the Russian Far East around 2004, 2 years after the emergence of CRF63_02A6 HIV-1 in Siberia [4].

In the group of 18 patients from our study sample in whom CRF173_63A6 HIV-1 was isolated, 6 individuals were infected sexually, and 11 by using injected drugs. With the exception of one case of vertical mother-to-child transmission in 2011, there was no epidemiological link between these patients. For nine patients, HIV was diagnosed from 2011 to 2015, and eight individuals were diagnosed from 2018 to 2024. Eleven out of eighteen patients from this group live in the Oblutchensky district in the JAR. The Oblutchensky district is the most disadvantaged area in the JAR, where 34% of all patients with HIV identified in the JAR over the entire period of the HIV spread are registered; the HIV attack rate per 100,000 population (638.4) for the Oblutchensky district is 2.2 times higher than the regional average.

**Figure 2:**
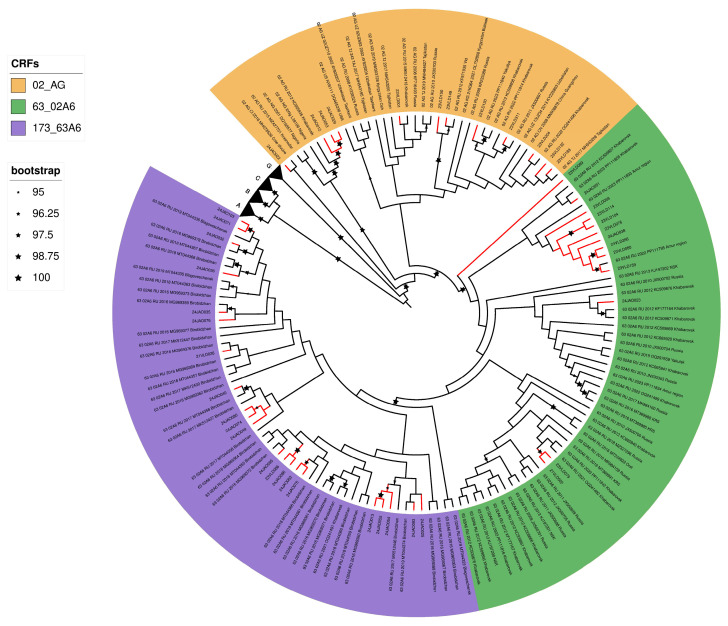
Maximum likelihood phylogenetic tree (IQ-Tree) of HIV-1 pol gene fragments (position 2262 to 3300 relative to HXB2) sequences. Black branches are sequences taken from GenBank and red branches are ones we received.

Analysis of HIV-1 pol sequences previously deposited in GenBank revealed a phylogenetic cluster combining the 18 CRF173_63A6 genovariants studied by us with 36 other HIV-1 samples (Figure 2). Of the 36 HIV-1 samples, 32 samples were isolated and deposited from 2016 to 2019 in the JAR, 3 in 2019 in Blagoveshchensk, and 1 in 2021 in Khabarovsk. An analysis of epidemiological data from the JAR residents infected with CRF173_63A6 shows that this virus is actively spreading in the JAR among PWID and is transmitted through sexual contact.

## 4. Discussion

The global spread of HIV-1 in Russia since the early 1990s has been associated with an active increase in the number of people who inject drugs [21,22,23]. In many areas of the country, HIV transmission in the key group of PWID dominated until 2014–2016. After 2010, the number of people using injectable drugs in Russia began to decrease, but at the same time, the consumption of more accessible synthetic or pharmacy drugs increased sharply, and the spread of HIV began to occur in the key group of PWID and their sexual partners [24]. Currently in Russia, the transmission of HIV infection is predominantly through heterosexual contact, but unprotected sexual contact can also be provoked by the use of smoking narcotic mixtures and synthetic drugs [25].

The circulation of different genetic variants of HIV and the practice of risk behavior in relation to infection are necessary and sufficient conditions for the emergence of new recombinant forms of HIV.

Based on the phylogenetic analysis and analysis of the recombination structure complete viral genomes, our study describes a new second-generation circulating recombinant form of HIV-1 that has spread in the Far Eastern Federal District of Russia. The second-generation recombinant form of the HIV-1 CRF173_63A6 subtype, which developed from the Russian A6 subtype and the CRF63_02A6 recombinant virus responsible for HIV outbreaks among PWID in the Siberian region, is described. Epidemiological data of the patients with CRF173_63A6 viruses indicate that this subtype has begun to spread among injecting drug users (IDUs). Recently described by us, HIV-1 CRF157_A6C, CRF147_A6B, and CRF133_A6B have also been identified among IDUs and their sexual partners [9,26,27].

The research data once again proves the significant contribution of the key group of PWID not only to the development of local Russian HIV epidemics, but also to the change in the characteristics of the circulating virus population [9,26,27].

The increase in the registered cases of various HIV-1 URFs and the spread of the emerging recombinant HIVs emphasizes the importance of strengthening infection prevention measures, including HIV reinfection in the key groups. Monitoring the spread of new emerging HIV is essential for understanding the evolution and patterns of change in the molecular epidemiology of the virus.

## Data Availability

The original contributions presented in this study are included in the article/Supplementary Materials. Further inquiries can be directed to the corresponding author.

## Supplementary Materials

The following supporting information can be downloaded at: https://www.mdpi.com/article/10.3390/pathogens14090836/s1, Figure S1: Dated phylogenetic analysis of NLFG HIV-1 subtype CRF63_02A6 and CRF173_63A6 genomic sequences; Table S1: Demographic and clinical characteristics of the patients who had novel CRF173_63A6 infection.

## Abbreviations

The following abbreviations are used in this manuscript:

HIV	Human immunodeficiency viruses
JAR	Jewish Autonomous Region
AIDS	Acquired Immunodeficiency Syndrome
NFLG	Near full-length genome
CRF	Circulating recombinant form
PWID	People who inject drugs
IDU	Injecting drug user
URF	Unique recombinant form
RIP	Recombinant identification program
tMRCA	Time to the most recent common ancestor
