# Supplementary material for: Characterization of a New HIV-1 Second-Generation Circulating Recombinant Form CRF173_63A6 in the Jewish Autonomous Region of Russia
Source: Pathogens. 2025 Aug 22;14(9):836. doi: 10.3390/pathogens14090836 (PMC12472342; doi:10.3390/pathogens14090836)
Supplement: Supplementary file 1 [file pathogens-14-00836-s001.zip › Table S1 Revised1.pdf]

Table S1. Demographic and clinical characteristics of the patients who had novel 173_63A6 infection

Sample ID	The accession numbers	Date of isolation	Region	Gender	Age	Route of infection	First HIV-positive visit	Viral load (copies/mL)	CD4 cell count (cells/mm³)	Stage of HIV infection	ART regimen	HIV-1 subtype
24JAR032	PQ523366	2024	Obluchye	M	48	PWID	2015	nd	832	3	TDF+3TC+EFV	173_63A6
24JAR035	PQ523367	2024	Birobidzhan	M	45	PWID	2024	32000	523	3	ARV-naive	173_63A6
24JAR036	PQ523368	2024	Obluchye	M	52	PWID	2018	150000	290	3	ARV-naive	173_63A6
24JAR049	PQ523369	2024	Obluchye	M	45	HET	2013	58	605	4A	TDF+3TC+EFV	173_63A6
24JAR054	PQ523370	2024	Obluchye	M	41	PWID	2018	nd	502	3	TDF+3TC+DTG	173_63A6
24JAR055	PQ523371	2024	Birobidzhan	F	45	HET	2024	580000	320	3	ARV-naive	173_63A6
24JAR071	PQ523372	2024	Dvurechye	M	55	HET	2019	nd	268	3	TDF+3TC+EFV	173_63A6
24JAR086	PQ5233731	2024	Obluchye	M	52	HET	2013	12200	307	4A	TDF+3TC+EFV	173_63A6
24JAR093	PQ523374	2024	Obluchye	F	63	HET	2022	58	335	4A	TDF+3TC+EFV	173_63A6
24JAR095	PQ523375	2024	Birobidzhan	F	46	PWID	2019	510	1206	3	TDF+3TC+EFV	173_63A6
21VLD026	PQ523377	2021	Artyom	M	45	PWID	2021	77926	224	4A	ARV-naive	173_63A6
24JAR103	PQ523376	2024	Birobidzhan	M	41	HET	2018	150000	10	4Б	TDF+3TC+DRV/r	173_63A6

This table represents the demographic and clinical characteristics of the study individuals with a novel 173_63A6 HIV-1 infection. For patients who had exposure to several antiretroviral (ART) regimens, all the regimens are listed in order as they were prescribed such as the top regimen represents the most recent.

Abbreviations: VLD: Vladivostok; JAR: Jewish Autonomous Region; M: male; F: female; nd: no data; PWID: people who injected drug; HET: heterosexual contact; ART: antiretroviral therapy; DRV: darunavir; DTG: dolutegravir; EFV: efavirenz; TDF: tenofovir; 3TC: lamivudine.
