# Supplementary figures and images for: Characterization of a New HIV-1 Second-Generation Circulating Recombinant Form CRF173_63A6 in the Jewish Autonomous Region of Russia
Source: Pathogens. 2025 Aug 22;14(9):836. doi: 10.3390/pathogens14090836 (PMC12472342; doi:10.3390/pathogens14090836)

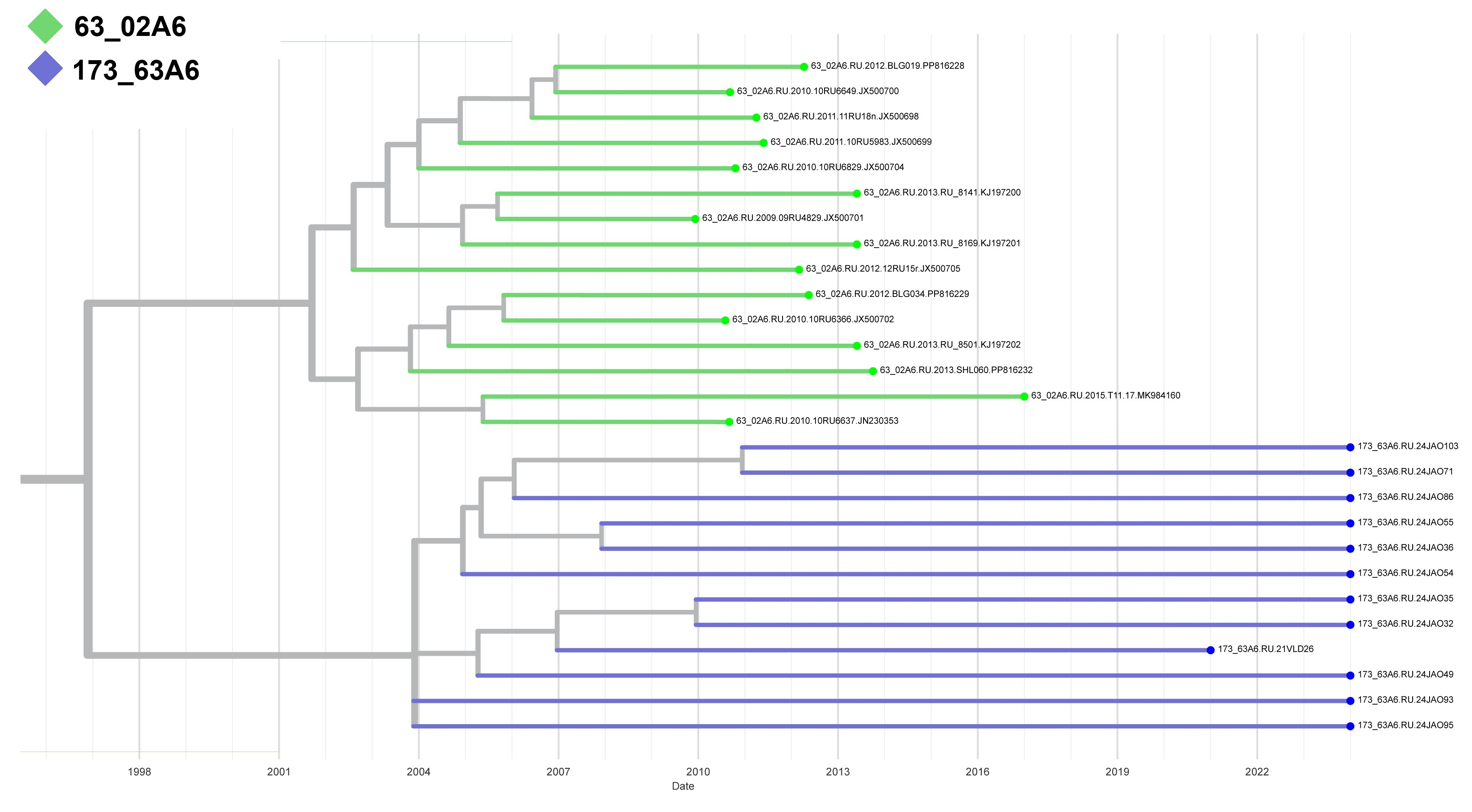

Supplement: Supplementary file 1 [file pathogens-14-00836-s001.zip › Figure S1.png]
